# A Non-Viral Plasmid DNA Delivery System Consisting on a Lysine-Derived Cationic Lipid Mixed with a Fusogenic Lipid

**DOI:** 10.3390/pharmaceutics11120632

**Published:** 2019-11-27

**Authors:** María Martínez-Negro, Natalia Sánchez-Arribas, Andrés Guerrero-Martínez, María Luisa Moyá, Conchita Tros de Ilarduya, Francisco Mendicuti, Emilio Aicart, Elena Junquera

**Affiliations:** 1 Departamento de Química Física, Facultad de Ciencias Químicas, Universidad Complutense de Madrid, 28040 Madrid, Spain; mmnegro@ucm.es (M.M.-N.); natsanch@ucm.es (N.S.-A.); aguerrero@quim.ucm.es (A.G.-M.); aicart@ucm.es (E.A.); 2 Grupo de Química Coloidal y Catálisis Micelar, Departamento de Química Física, Facultad de Química, Universidad de Sevilla, 41012 Sevilla, Spain; moya@us.es; 3 Departamento de Tecnología y Química Farmacéuticas, Facultad de Farmacia y Nutrición, Universidad de Navarra, Instituto de Investigación Sanitaria de Navarra (IdiSNA), 31080 Pamplona, Spain; ctros@unav.es; 4 Departmento de Química Analítica, Química Física e Ingeniería Química and Instituto de Investigación Quimica Andrés M. del Rio, Universidad de Alcalá, 28871 Alcalá de Henares, Spain; francisco.mendicuti@uah.es

**Keywords:** lysine-derived cationic lipid, plasmid DNA, lipoplex, transfection, protection, compaction, gene delivery, multilamellar aggregates, molecular dynamics

## Abstract

The insertion of biocompatible amino acid moieties in non-viral gene nanocarriers is an attractive approach that has been recently gaining interest. In this work, a cationic lipid, consisting of a lysine-derived moiety linked to a C_12_ chain (LYCl) was combined with a common fusogenic helper lipid (DOPE) and evaluated as a potential vehicle to transfect two plasmid DNAs (encoding green fluorescent protein GFP and luciferase) into COS-7 cells. A multidisciplinary approach has been followed: (i) biophysical characterization based on zeta potential, gel electrophoresis, small-angle X-ray scattering (SAXS), and cryo-transmission electronic microscopy (cryo-TEM); (ii) biological studies by fluorescence assisted cell sorting (FACS), luminometry, and cytotoxicity experiments; and (iii) a computational study of the formation of lipid bilayers and their subsequent stabilization with DNA. The results indicate that LYCl/DOPE nanocarriers are capable of compacting the pDNAs and protecting them efficiently against DNase I degradation, by forming L_α_ lyotropic liquid crystal phases, with an average size of ~200 nm and low polydispersity that facilitate the cellular uptake process. The computational results confirmed that the LYCl/DOPE lipid bilayers are stable and also capable of stabilizing DNA fragments via lipoplex formation, with dimensions consistent with experimental values. The optimum formulations (found at 20% of LYCl content) were able to complete the transfection process efficiently and with high cell viabilities, even improving the outcomes of the positive control Lipo2000*.

## 1. Introduction 

Therapeutic agents capable of replacing/silencing damaged DNA are one of the principal areas of research in the branch of medicine known as gene therapy [1,2,3]. The development of biocompatible material-based non-viral vectors is a promising approach for the treatment of genetic diseases [4,5,6]. The design of nanocarriers able to compact, protect, transport, and deliver nucleic acids into target cells with high efficiency and minimum toxicity is the major challenge in this scientific area. 

Numerous artificial systems formed by lipids [7,8,9], polymers [10,11,12], dendrimers [13,14], carbohydrates [15,16], cyclodextrins [17,18], polypeptides [19,20], and nanoparticles [21,22] (among others) have been studied to avoid the adverse effects produced by viral vectors in biological media, such as immunogenicity and inflammatory responses [23,24]. In particular, cationic lipids have been investigated in detail as useful tools [25,26] because they present low toxicity and are easily synthesized, as well as establish strong electrostatic interactions with DNA/RNA double strains through their positive charges affording lipoplexes. Furthermore, the nanometer size and net positive charge of these complexes favors their internalization across the cell membrane, allowing the delivery of the cargo in the cellular cytoplasm and resulting in moderate-to-high transfection efficacy. On the other hand, amino acids have an important role in cell life as structural blocks of proteins. They are considered essential in metabolic processes and as regulators of gene expression. In fact, they can form gene carriers alone, such as peptide-based vectors [19,27,28,29] like the well-known poly-l-lysine (PLL) vector, the first cationic peptide ever used for the delivery of nucleic acids [30]. The repeated lysine units endow this vector with superior biodegradability and low toxicity and, hence, it soon began to be used for in vivo applications [31,32]. However, its poor circulatory half-life leads to inefficient delivery of the cargo. Since the presence of amino acid residues improves the biocompatibility and cationic lipids are able to establish electrostatic interactions with DNA, some studies have been reported on their combination to design and synthesize alternative candidates for plasmid DNA/small interfering RNA nanocarriers [33,34,35,36]. Mono- [37,38,39], di- [38,40,41], tri- [38,41], or multi-peptide [41] cationic lipid systems have been used in in vitro studies for DNA delivery. In mono-histidine cationic lipids [38] and lysine cationic lipid assemblies [34], moderate-to-high transfection efficiencies have been achieved. Koloskova et al. reported an aliphatic lipopeptide as an efficient delivery and silencing gene vector [41]. It has been already reported that gene delivery systems formed by amino acid-based cationic lipids are internalized by habitual endocytic uptake pathways. Thus, the incorporation of amino acid moieties to the head groups of cationic lipids does not seem to change the cellular uptake mechanism, but it can improve the intracellular delivery [33,42,43,44,45].

In previous studies, we have also demonstrated the advantages of using gene carriers based on amino acid components within a gemini-type lipid [46,47]. Generally speaking, those works have demonstrated that the use of this kind of molecular platforms is a safe option for biological studies. However, none of them included computational studies to understand the contribution of the amino acid residues placed on the cationic head group of the lipid structure toward the condensation of DNA and stabilization of the lipoplexes.

In this work, we present a monovalent cationic lipid as a promising alternative to complex plasmid DNA. To improve its biocompatibility, a lysine-derived residue was introduced in the cationic head. Thus, mixtures of this single-chain cationic lipid, (*S*)-5-acetamido-6-(dodecylamino)-*N,N,N*-trimethyl-6-oxohexan-1-ammonium chloride (LYCl), and a well-known fusogenic helper lipid, 1,2-dioleoyl-*sn*-glycero-3-phosphatidyl ethanol amine (DOPE), were used to compact, protect, and transfect two different plasmid DNAs: those encoding the green fluorescent protein GFP (pEGFP-C3) and luciferase (pCMV-Luc). Our approach is based on complementary experimental and computational studies that provide a global vision of these systems. Accordingly, compaction of the plasmids was evaluated by zeta potential analysis and agarose gel electrophoresis. Structural characterization was performed by small-angle X-ray scattering (SAXS) and cryo-transmission electronic microscopy (cryo-TEM). Molecular mechanics (MM) and molecular dynamics (MD) calculations supported the formation and stability of complexes formed by a mixture of lipids and DNA. Biological experiments with the COS-7 cell line were performed to demonstrate the versatility, efficacy, and safety of the present vector. To confirm the delivery of nucleic acids into the cellular cytoplasm, transfection efficiency studies were carried out by fluorescence assisted cell sorting (FACS) and luminometry. The biocompatibility of the nanocarrier reported in this work was further assessed by cell viability assays. Another important aspect related to plasmid protection against degradation in biological fluids was evaluated by agarose gel electrophoresis upon previous incubation of the lipoplexes in the presence of DNAse I. The present wide experimental and computational study demonstrates the potential of LYCl/DOPE-pDNA lipoplexes as efficient nucleic acid nanocarriers for future in vivo applications.

## 2. Materials and Methods 

### 2.1. Materials

**Figure 1:**
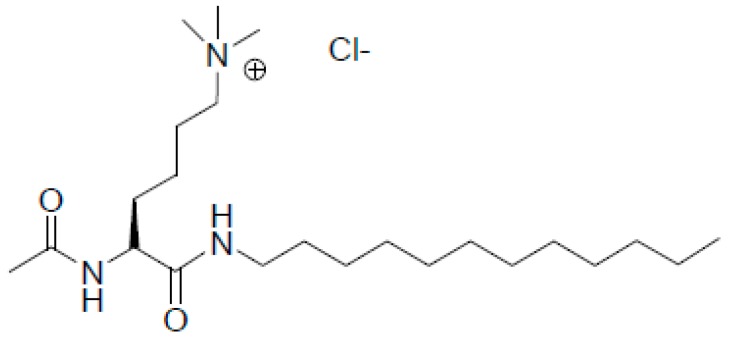
Molecular structure of the amino acid-based cationic lipid LYCl.

The lysine-derived surfactant (*S*)-5-acetamido-6-(dodecylamino)-*N,N,N*-trimethyl-6-oxohexan-1-ammonium chloride, LYCl (Figure 1), has been previously reported. The details of this synthesis and wide physicochemical characterization in aqueous solution have been published elsewhere [48].

Zwitterionic lipid 1, 2-dioleoyl-*sn*-glycero-3-phosphatidyl ethanol amine (DOPE) was purchased with the highest purity from Avanti Polar Lipids, Inc. (Alabaster, AL, USA). The sodium salt of calf thymus DNA (ctDNA), as provided by Sigma-Aldrich (St. Louis, MO, USA), was used as linear DNA to determine the effective charge of the cationic lipid (LYCl). Plasmid DNA pEGFP-C3 (4700 bp) encoding GFP was extracted from competent *Escherichia coli* bacteria previously transformed with pEGFP-C3. The extraction was carried out using a GenElute HP Select Plasmid Gigaprep Kit (Sigma Aldrich). Plasmid DNA pCMV-Luc VR1216 (6934 bp) encoding luciferase (Clontech, Palo Alto, CA, USA) was amplified in *E. coli* and isolated and purified using a Qiagen Plasmid Giga Kit (Qiagen GMBH, Hilden, Germany). All the reagents and solvents were of the highest grade commercially available and used without further purification.

### 2.2. Preparation of Lipoplexes

Lipid mixtures were prepared by dissolving appropriate amounts of cationic LYCl and zwitterionic DOPE to fit the selected molar fractions of the cationic lipid (α) following a procedure previously described [49,50]. Lipoplexes were finally prepared by mixing appropriate amounts of the pDNAs and mixed lipid suspensions. The optimum pDNA concentrations were chosen for each experimental technique, as follows: 0.1 mg/mL for zeta potential, 0.1 mg/mL for gel electrophoresis experiments, 1 mg/mL for cryo-TEM, 200 μg/capillary (~5 mg/mL) for SAXS, and 1 μg/well (1 μg/mL) for biological studies.

### 2.3. Zeta Potential and Particle Size 

The electrophoretic mobility, from which the zeta potential of the nanoaggregates was determined, was measured by a phase analysis light scattering technique (Zeta PALS, Brookhaven Instruments Corp., Holtsville, NY, USA). On the other hand, the particle size was determined using a particle analyzer (Zeta Nano Series; Malvern Instruments, Barcelona, Spain). The experimental conditions and preparation of the samples have been described in detail elsewhere [50]. The experiments were planned at different molar fractions (α) of the cationic lipid in the LYCl/DOPE mixtures by measuring the electrophoretic mobility and particle size as a function of the total mixed lipid/DNA mass ratio ((m_L_^+^ + m_L_^0^)/m_DNA_). Each zeta potential and particle size data point represent the average of 50 and 30 independent measurements, respectively.

### 2.4. Gel Electrophoresis 

#### 2.4.1. DNA Compaction Assay

To analyze the pDNA compaction by the LYCl/DOPE mixed lipids, agarose gel electrophoresis experiments were carried out using a Gel Doc XR instrument (Bio-Rad, Hercules, CA, USA). Details of the experimental conditions were reported previously [51].

#### 2.4.2. DNA Protection Assay

Following a procedure described elsewhere [51], a Gel Doc XR instrument (Bio-Rad) was also used for gel electrophoresis experiments to evaluate the pDNA protection upon previous incubation of LYCl/DOPE-pDNA lipoplexes in DNase media. Untreated DNA was used as a control to evaluate the integrity of the plasmid at each composition.

### 2.5. Small Angle X-Ray Scattering

SAXS diffractograms were registered at the ALBA Synchrotron (Barcelona, Spain, beamline BL11) following an experimental procedure reported elsewhere [52]. A Quantum 210 r CCD detector was used to detect the scattered X-rays, which were further converted into one-dimensional scattering by radial averaging, and plotted as a function of the momentum transfer vector (q). SAXS experiments were performed for LYCl/DOPE-pDNA lipoplexes at different effective charge ratios (*ρ*_eff_) of the lipoplexes and at several cationic lipid compositions of the mixed lipids (α).

### 2.6. Cryo-TEM

According to the standard protocol [53,54,55], micrographs of LYCl/DOPE-pDNA lipoplexes were obtained at different effective charge ratios (*ρ*_eff_) of the lipoplexes and at two cationic lipid compositions of the mixed lipids (α). For that purpose, a JEM 2011 microscope (Jeol, Peabody, MA, USA) was operated at 200 kV under low-dose conditions and with different degrees of defocus (500–700 nm). The Digital Micrograph software was used to process and analyze the CCD images of the LYCl/DOPE-pDNA lipoplexes.

### 2.7. Cell Culture

African green monkey kidney (COS-7) cells (American Type Collection, Rockville, MD, USA) were maintained at 37 °C under 5% CO_2_ in complete medium.

### 2.8. In Vitro Transfection Efficiency

The transfection efficiency of LYCl/DOPE-pDNA lipoplexes was evaluated by two methods, luminometry (pDNA encoding luciferase) and FACS (pDNA encoding GFP) 48 h after transfecting 1 µg of pDNA/well. In both cases, 48 well-plates are used for seeding 100,000 cells/well. The measurements were carried out in triplicate from three independent cultures and using Lipofectamine 2000 (Lipo2000*) as a positive control. Conditions: 1.5 μL of Lipo2000*/μg of DNA.

#### 2.8.1. Luminometry 

The luciferase activity was determined using the luciferase assay reagent (Promega, Durham, NC, USA) and a luminometer (Sirius-2, Berthold Detection Systems, Innogenetics, Diagnóstica y Terapéutica, Barcelona, Spain), following the procedure and experimental conditions previously described [52]. The protein content was measured using the DC protein assay reagent (Bio-Rad, Hercules, CA, USA), from which the data were obtained in RLU/mg protein and converted to ng of luciferase/mg of protein using a standard calibration curve.

#### 2.8.2. FACS 

FACS analysis was performed using a Calibur 345 flow cytometer and the BD CellQuest^TM^ Pro software (BD Bioscience, San Jose, CA, USA). Details of the process and apparatus are provided elsewhere [52]. Using the FlowJo LLC data software, the transfection efficiency was determined from the percentage of GFP cells observed (% GFP) and the average of fluorescence intensity per cell (mean fluorescence intensity, MFI).

### 2.9. Cell Viability

The cytotoxicity of the lipoplexes was evaluated by the Alamar Blue assay 48 h after transfecting 1 µg of pDNA/well in 48 well-plates, as previously reported [51,52]. The absorbance values of treated and untreated cells at 570 and 600 nm are related through the expression: (A_570_ − A_600_)_treated cells_ × 100/(A_570_ − A_600_)_control cells_, from which the percentage of cell viability was determined. Each sample was measured in three independent wells and Lipo2000* was used as the positive control. Conditions: 1.5 μL of Lipo2000*/μg of DNA. 

### 2.10. Computational Studies

Molecular mechanics (MM) and molecular dynamics (MD) calculations were performed to study the stability and forces responsible for the formation of lipid bilayers at two different compositions of LYCl and DOPE, which were built step by step by adding the different LYCl and DOPE units, as described in the Supplementary Material (SM). The interaction of these lipid bilayers with two β-DNA helical fragments in a periodic box of explicit water is also described in this work. For the calculations, Sybyl X-2.0 and Tripos Force Field [56] were used. The charges of the DNA fragments (consisting of twelve nucleotides with CGCGAATTCGCG sequence in a composition similar to that of DNA calf-thymus), neutral DOPE, and the cationic LYCl lipid were determined by the Gasteiger and Marsili method [57,58]. Water solvation was performed using the Molecular Silverware algorithm (MS) and periodic boundary conditions (PBC) were also used [59]. A relative permittivity of ε = 1 was employed for electrostatic contributions in the presence of water. The non-bonded cut-off distances for MM, as well as for MD, were set at 12 Å. Optimizations were carried out by a simplex algorithm, and the conjugate gradient was used as the termination method with gradients of 1.5 Kcal/mol Å for the MM calculations [60,61]. Procedures and protocols for the 1 ns Molecular Dynamics trajectories on lipid bilayers/DNA lipoplexes were also described in the SM. 

## 3. Results and Discussion 

### 3.1. Electrochemical and Structural Characterization of LYCl/DOPE-pDNA Lipoplexes

In the field of cationic lipofection, the design and synthesis of lipid-based vectors capable of compacting and protecting plasmid DNAs by forming positively charged lipoplexes able to cross the negatively charged cellular membrane is of utmost importance, as well as the safe and efficient delivery of genetic material inside the cytoplasm. Therefore, the starting point of all lipofection studies should be the determination of the effective charges of both members of the lipoplex (lipid vector and plasmid), which do not necessarily have to be equal to the nominal ones, as demonstrated by us in previous works [50,62]. For that purpose, we have reported a protocol (fully described elsewhere [46,49,63] and summarized in the SM) based on a wide electrochemical study that uses both zeta potential and agarose gel electrophoresis experiments to determine the electroneutrality conditions of lipoplexes ${\left(m_{L}/m_{\mathrm{DNA}}\right)}_{\phi }$ and, subsequently, the effective charges of both the cationic lipid $\left(q_{{}_{\mathrm{eff},\;\mathrm{LYCl}}}^{+}\right)$ and pDNA $\left(q_{{}_{\mathrm{eff},\;\mathrm{pDNA}}}^{-}\right)$. 

**Figure 2:**
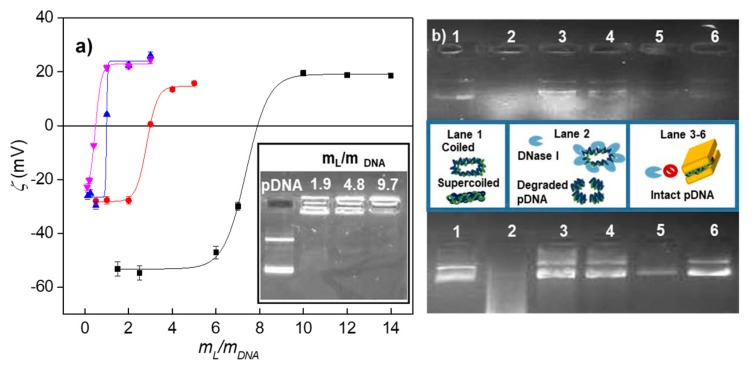
(**a**) Plot of the zeta potential as a function of the $m_{L}/m_{\mathrm{DNA}}$ mass ratio of LYCl/DOPE-DNA lipoplexes, constituted by ctDNA at a molar composition of the cationic lipid in the mixed lipids of α = 0.5 (black line) and with pDNA at α = 0.2, 0.5, and 0.7 (red, blue, and pink lines). Inset: agarose gel electrophoresis of LYCl/DOPE-DNA lipoplexes at several $m_{L}/m_{\mathrm{DNA}}$ mass ratios and α = 0.5. Free pDNA (lane 1) was used as the control. Errors are within ±5%. (**b**) Protection assays of pDNA against degradation by DNase I: Top, pEGFP-C3 plasmid; Bottom, pCMV-Luc plasmid. In both experiments (a schematic drawing has been included): lane 1, pDNA; lane 2, pDNA-DNase I; and lanes 3–6, LYCl/DOPE-pDNA lipoplexes at different molar compositions of the cationic lipid in the mixed lipids (α = 0.2 in lanes 3 and 4, and α = 0.5 in lanes 5 and 6) and at different effective charge ratios of the lipoplex (*ρ*_eff_ = 4 in lanes 3 and 5, and *ρ*_eff_ = 10 in lanes 4 and 6).

Figure 2a shows both studies (the zeta potential curves in the main figure and agarose gel electrophoresis in the inset) for the LYCl/DOPE-pDNA lipoplexes studied in this work at different LYCl molar compositions. The zeta potential data plotted against the $m_{L}/m_{\mathrm{DNA}}\left(=(m_{L^{+}}+m_{L^{0}})/m_{\mathrm{DNA}}\right)$ mass ratios (where $m_{L^{+}}$, $m_{L^{0}}$, and $m_{\mathrm{DNA}}$ are the mass of the LYCl cationic lipid, DOPE helper lipid, and plasmid DNA, respectively) display the typical sigmoidal profile, where charge inversion can be observed at specific ${\left(m_{L}/m_{\mathrm{DNA}}\right)}_{\phi }$ values corresponding to the electroneutrality ratios (see Table S1). From these ratios and using the procedure described above, the following effective charges were calculated as $\left(q_{{}_{\mathrm{eff},\;\mathrm{LYCl}}}^{+}=+0.45\;\pm \;0.05\right)$ and $\left(q_{{}_{\mathrm{eff},\;\mathrm{pDNA}}}^{-}=-0.24\;\pm \;0.05\right)$ (see Table S2). These results indicate that both the cationic lipid and plasmid show net positive and negative charges, respectively, that are quite different from their nominal ones: LYCl exhibits only ~45% of its positive nominal charge (+1), while pDNA displays on average only ~12% of its negative nominal charge (−2/bp). This behavior has also been reported for other lipoplexes [46,52], revealing that the supercoiled conformation adopted by the plasmid under physiological conditions retains a significant percentage of Na^+^ counterions associated to the phosphate groups of pDNA upon lipoplex formation, while the rest are expelled to the bulk contributing to a clear entropy gain. This factor, together with strong electrostatic interactions, is considered the main driving force toward lipoplex formation. On the other hand, the inset of Figure 2a shows the corresponding agarose gel electrophoresis experiment. The two fluorescent bands observed in the first lane correspond to the coiled and supercoiled forms of the anionic plasmid DNA, used as the control, moving towards the anode. Accordingly, the absence of these fluorescent bands in the other lanes indicates that pDNA has been efficiently compacted by the cationic nanovector, leading to charge inversion that subsequently induces the cationic lipoplex to remain in the catode, i.e., the lipoplex remains in the well of the gel where fluorescence emission of the intercalated probe is observed. The transition between these two electrophoretic patterns provides a range of mass ratios where electroneutrality is reached. As can be observed in the figure, pDNA is already fully compacted by LYCl/DOPE mixed lipids at $m_{L}/m_{\mathrm{DNA}}=1.9$ and α = 0.5, in good agreement with the zeta potential study reported in the main figure. Once the effective charges are known, the effective charge ratios (*ρ*_eff_) of the LYCl/DOPE-pDNA lipoplexes can be easily determined through the following relation:

(1) $$\rho_{\mathrm{eff}}=\frac{n^{+}}{n^{-}}=\frac{q_{\mathrm{eff},\mathrm{LYCl}}^{+}(m_{\mathrm{LYCl}}/M_{\mathrm{LYCl}})}{q_{\mathrm{eff},\mathrm{pDNA}}^{-}(m_{\mathrm{pDNA}}/M_{\mathrm{pDNA}})}$$

where n^+^ and n^−^ are the positive and negative charges, respectively, and M_LYCl_ and M_pDNA_ are the molecular weight of LYCl and pDNA, respectively.

Furthermore, the cationic gene vector is required not only to compact the pDNA but also to efficiently protect it against the degrading action of DNase I present in the serum. Figure 2b show the protection assays carried out for the lipoplexes consisting of LYCl/DOPE mixed lipids at two molar compositions (α = 0.2 and 0.5) and two plasmids (pEGFP-C3 at the top and pCMV-Luc at the bottom) at two effective charge ratios (*ρ*_eff_ = 4 and 10). This gel electrophoresis study involved also two controls: (i) the naked plasmid, loaded in the first lane, and (ii) degraded pDNA by previous digestion with DNase I, loaded in the second lane. Notice the characteristic fluorescent bands of pDNA in lane 1 and the corresponding absence of bands in the second lane. Consequently, the presence of DNA bands on the other lanes (3–6) evidences that the nanovector has adequately compacted the plasmid by forming a lipoplex thus preventing the action of the degrading enzyme. It is remarkable that this protection is efficient for both plasmids under all the studied experimental conditions (α and *ρ*_eff_ values).

The structural characteristics of the lipoplexes, such as the size, polydispersity, structure, and morphology, are known to play as well a key role in their potential success as efficient gene vectors. For that reason, all these aspects were evaluated by Dynamic Light Scattering (DLS) measurements (coupled to the electrophoretic mobility-zeta potential technique) and SAXS and cryo-TEM analyses. Table S3 summarizes the hydrodynamic size (D_h_) and polydispersity index (PDI) values for the lipoplexes composed of LYCl/DOPE mixed lipids (at two different molar fractions) and two different pDNA plasmids (pEGFP-C3 and pCMV-Luc) at two effective charge ratios of the lipoplex. Sizes in the range of 150–200 nm and low PDIs, such as those reported here, are believed to be adequate to guarantee efficient crossing through the cell membrane.

**Figure 3:**
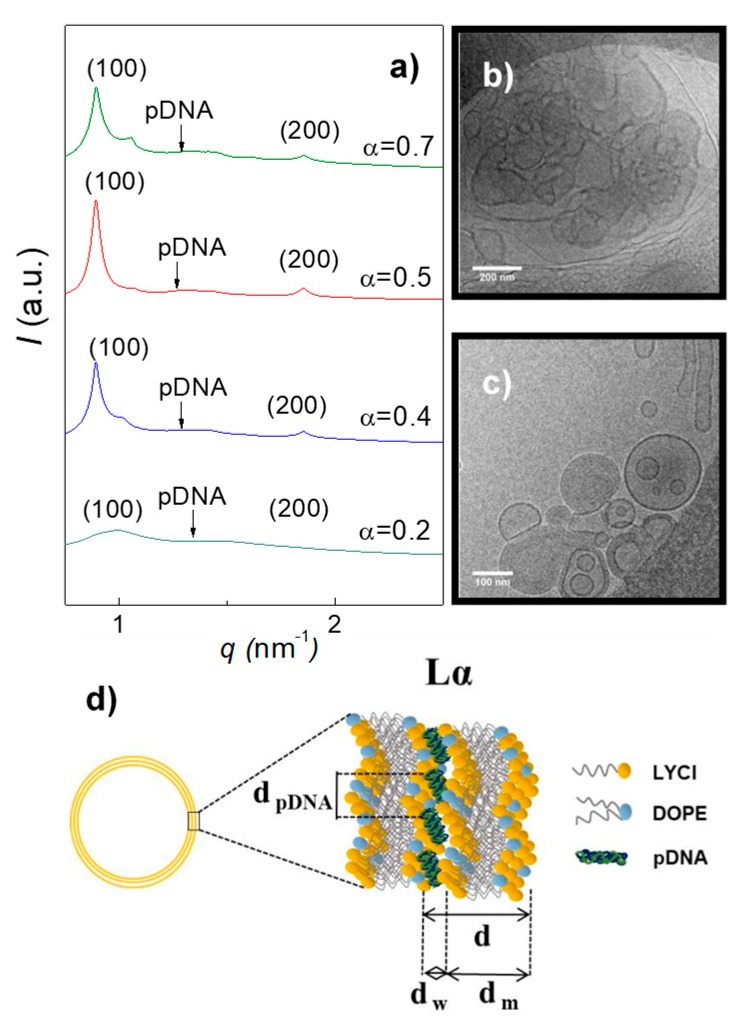
(**a**) SAXS diffractograms of LYCl/DOPE-pDNA lipoplexes at an effective charge ratio of *ρ*_eff_ = 4 and different molar compositions (α). (**b**,**c**) A selection of cryo-TEM micrographs of LYCl/DOPE-pDNA lipoplexes at *ρ*_eff_ = 4 and molar compositions of the cationic lipid in the mixed lipids of (**b**) α = 0.2 and (**c**) α = 0.5. Scale bars are (**b**) 200 nm and (**c**) 100 nm. (**d**) 3D scheme of the L_α_ multilamellar lyotropic liquid crystal phase.

The combination of SAXS and cryo-TEM analyses has become a powerful tool to obtain structural information for this type of systems. These experiments were carried out for LYCl/DOPE-pDNA lipoplexes at different molar compositions of the mixed lipids (α) and effective charge ratios of the lipoplex (*ρ*_eff_). Figure 3 collects the SAXS diffractograms (panel a, plots of intensity vs momentum transfer vector (q) and a selection of cryo-TEM micrographs (panels b and c) at *ρ*_eff_ = 4, as an example. Further SAXS data and cryo-TEM micrographs are provided in Table S4 and Figure S1. Indexation of the Bragg peaks in all the diffractograms provided a clear hkl sequence (100, 200, etc.) corresponding to the well-known L_α_ lyotropic crystal phase. L_α_ is a multilamellar phase, where each lamella recalls the characteristic structure of the cell membrane. It can be understood as a sandwich-type structure where bilayers of the LYCl/DOPE mixed lipid, positively charged, alternate with aqueous monolayers, where the anionic plasmid DNA is electrostatically compacted (see the schematic drawing in panel d of Figure 3). This aggregation and compaction pattern is also observed on the cryo-TEM micrographs shown in panels b and c of Figure 3 (and those provided in Figure S1). It should be noted the presence of cluster-type shapes (CT-type) as clear projections of these multilamellar phases. The interlamellar periodic distance (d), one of the characteristic structural parameters of this L_α_ phase (see Figure 3d), can be obtained from the q factors at which the Bragg peaks are found in the diffractograms ($d=2\pi n/q_{n00}$, where n is the diffraction order) and, less accurately, from the cryo-TEM micrographs. From the SAXS data, an average value of d = 6.8 ± 0.7 nm was obtained for the LYCl/DOPE-pDNA lipoplexes in this work (see Table S4). This value, which is almost constant (within experimental uncertainty) at low-to-moderate compositions (α ≤ 0.5), is consistent with those previously reported [51,64] for other lipoplexes consisting of plasmid DNA and a lipidic vector formed by DOPE (two C_18_ alkyl chains) and a cationic lipid with an alkyl chain of similar length. It seems that *ρ*_eff_ does not have a marked effect on d either. As can be seen in the scheme reported in Figure 3d, d can be expressed as the sum of the thicknesses of the aqueous monolayer (d_w_) and lipid bilayer (d_m_), last one estimated using either Tanford’s model [65,66,67] (d_m_ ~4.8 nm) or from the cryo-TEM micrographs (d_m_ ~4.5 nm). Accordingly, an aqueous monolayer of d_w_ ~2.0–2.3 nm thickness is expected for the LYCl/DOPE-pDNA lipoplexes, perfectly adequate to electrostatically house and compact pDNA. On the other hand, the broad Bragg peak observed in the middle of the diffractograms, indexed to pDNA–pDNA correlations, allows to determine the distance between pDNA supercoils in the aqueous monolayer (d_pDNA_ = 2π/q_pDNA_). As can be seen in Table S4, this distance does not seem to depend on neither α nor *ρ*_eff_ at low-to-moderate LYCl contents in the lipidic mixture (α ≤ 0.5).

Atomistic simulation methods have been widely used to study structural, dynamic, and biological phenomena occurring in lipid bilayers. An interesting review on these methods has been recently published [68]. However, the interactions between DNA and mixed lipid bilayers while forming lipoplexes and their structural details, which seem to play crucial roles in DNA compaction and transfection processes, have been less studied [69,70,71,72,73]. In this work, we have carried out a computational study of these interactions, which are proposed to proceed in two stages. Firstly, two lipid bilayers constituted by LYCl and DOPE at different compositions were generated (denoted 4LYCl/9DOPEDIM and 12LYCl/9DOPEDIM) and subsequently made interact with two DNA fragments located at both sides of the lamellar structure. The building protocols for the two mixed lipid bilayers are fully described in the SM (that also includes Figures S2–S16), while the formation and stabilization of the 3D lipid bilayer-DNA lipoplexes is summarized as follows.

The 3D lipid bilayer previously generated was located, as shown in Figure S14 for 12LYCl/9DOPEDIM as an example, with its center of mass at the origin of a coordinate system between two symmetrically located and properly oriented DNA fragments. The DNAs were initially placed at distances where they barely interact with the lipid bilayer and then simultaneously brought closer along the major groove, in 0.33 Å steps along the y coordinate, i.e., from y = +50 to +30 Å and from y = −50 to −30 Å, respectively. Every structure generated was solvated (MS and PBC), optimized (gradient 1.5 kcal/mol Å), and analyzed. The most stable lipid bilayer/DNA_2_ lipoplex structures (MBE) generated were optimized once again (gradient 0.5 kcal/mol Å) and used as the starting conformations for 1-ns MD simulations in the presence of water, following the strategy briefly described in the SM, which is similar to that used in previous reports [74,75,76,77]. 

**Figure 4:**
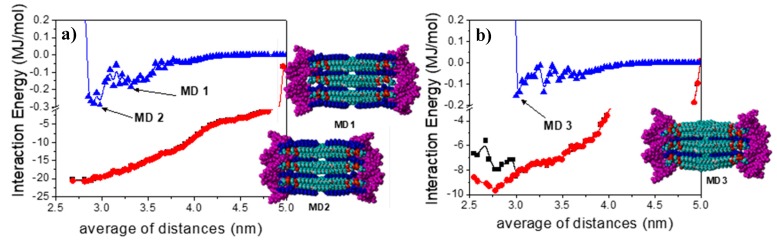
(**a**) Total interaction energy (black), and electrostatic (red) and van der Waals (blue) contributions for the DNA fragments and 12LYCl/9DOPEDIM lipid bilayer versus the average of the d_1_ and d_2_ distances between the center of masses of DNA and 12LYCl/9DOPEDIM (see Figure S14). (**b**) Idem for the 4LYCl/9DOPEDIM containing lipoplex. The insets are views (from the x axis) of the MD1, MD2, and MD3 MBE lipoplex structures obtained by MM calculations (DNA fragments in magenta).

Figure 4 shows the total 3D lipid bilayer/DNA_2_ interaction energy and van der Waals and electrostatic contributions as a function of the averaged d_1_ and d_2_ distances between the centers of mass of the DNA fragments and the lipid bilayers (12LYCl/9DOPEDIM or 4LYCl/9DOPEDIM), as shown in Figure S14. The lipid bilayer/DNA_2_ total interaction energy, depicted in Figure 4, decreased with the approaching DNA fragments. The largest contribution to this reduction was from electrostatic interactions. The van der Waals forces also decreased, becoming repulsive at distances smaller than ~3.0 nm. The local minima structures, denoted MD1 and MD2 for (12LYCl/9DOPEDIM)(DNA)_2_ and MD3 for (4LYCl/9DOPEDIM)(DNA)_2_, were used to perform MD simulations, as stated before. Figure S15b also depicts the total energy and van der Waals and electrostatic non-bonded contributions, as well as the strain energy for DNAs and the lipid bilayer separately (see Figure S15c) as a function of the same averaged distances. Similarly, the total energy decreased with the average of the distances, to then increase at distances shorter than ~3.0 nm. 

The main contribution to the total energy, also responsible for the system stabilization, arises from electrostatic forces. Van der Waals interactions, which are quantitatively smaller, slightly increased with the approaching DNA fragments. Something similar occurred for the bilayer and DNA fragment strain energies, which increased with the incoming DNAs and at distances smaller than 3.0 nm for both lipoplexes. The inset in Figure 4 illustrates the MD1 and MD2 structures for the (12LYCl/9DOPEDIM)(DNA)_2_ lipoplexes and MD3 for the (4LYCl/9DOPEDIM)(DNA)_2_ lipoplex employed in the MD simulations. 

**Figure 5:**
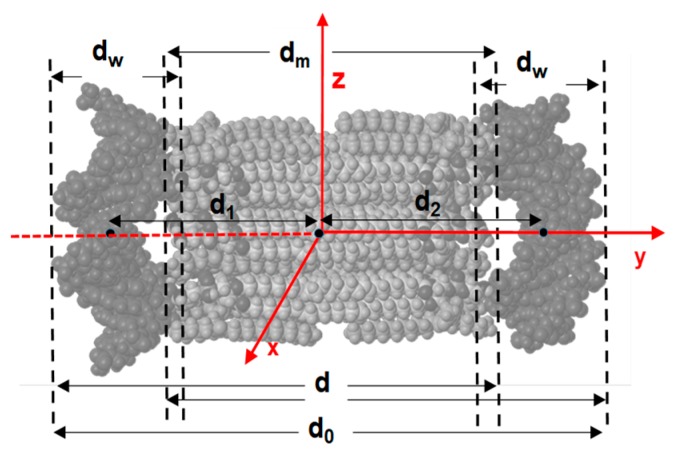
View of a lipoplex along the x axis. Several distances, calculated from the analysis of MD trajectories and used to analyze its structure, are shown. See the legend in Figure S16 for their definitions.

The evolution of the 3D lipid bilayer−DNA interaction energies and van der Waals and electrostatics contributions obtained from the analysis of the MD trajectories for the MD1, MD2, and MD3 structures also demonstrate the stability of the lipoplexes (see upper panels of Figure S16). In all studies, these interactions, which were initially favorable, remained as such during and at the end of the trajectories. Almost 100% of these interactions were due to electrostatic contributions, while van der Waals interactions were considerably smaller but also favorable. Stabilization, as expected, is more favorable for the lipoplex with the highest LYCl content. What is interesting is that no interaction existed or was repulsive between the DNA fragments located at the same distance as those in the nanocomplexes. This means that DNA fragments are only stabilized in the presence of cationic lipid bilayers upon forming supramolecular lipoplexes, in consistency with those evidences found in all the experimental studies, i.e., zeta potential and agarose gel electrophoresis (Figure 2a), and cryo-TEM and SAXS (Figure 3). The bottom panels in Figure S16 illustrate the evolution of several distances depicted in Figure 5 for the lipoplexes. 

**Figure 6:**
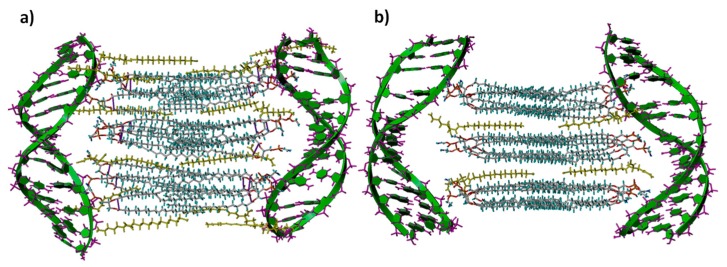
View of the lipoplexes averaged over the whole trajectory (ribbon mode for DNA): (**a**) (12LYCl/9DOPEDIM)(DNA)_2_ (denoted MD2) and (**b**) (4LYCl/9DOPEDIM)(DNA)_2_ (denoted MD3).

The evolution indicates that the DNA fragments and 3D bilayers remain at distances where they favorably interact during the whole trajectory. Table 1 collects these distances averaged along the whole MD trajectories. As can be seen in the table, the periodic distance (d) and thickness of the lipid bilayer (d_m_) are 6.4–6.7 nm and ~4.5 nm, respectively, for the different lipoplexes studied, in close agreement with the experimental values, obtained from both SAXS and cryo-TEM results (see the above discussion and also Table S4 of SM). Accordingly, d_w_ is ~2.1 nm, also in good agreement with the experimental range. This agreement is especially remarkable since SAXS technique allows us to obtain accurately d, while d_m_ is either estimated by cryo-TEM or with Tanford´s model, while MD calculations provide us these distances independently. Figure 6 shows the structures of the (12LYCl/3DOPEDIM)(DNA)_2_ and (4LYCl/9DOPEDIM)(DNA)_2_ lipoplexes averaged over the whole trajectory.

### 3.2. In Vitro Transfection and Cell Viability of LYCl/DOPE-pDNA Lipoplexes

**Figure 7:**
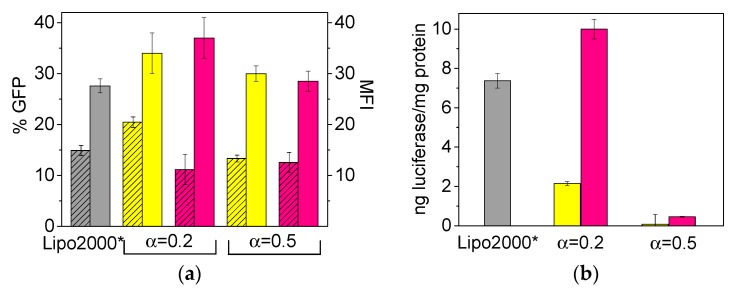
Transfection efficiency levels of LYCl/DOPE-pDNA lipoplexes in COS-7 cells at two molar compositions of the cationic lipid in the mixed lipids (α = 0.2 and 0.5) expressed (**a**) as % GFP (striped bars) and MFI (unstriped bars) for plasmid pEGFP-C3, and (**b**) as ng of luciferase/mg of protein for plasmid pCMV-Luc VR1216. All the experiments were performed with 10% serum (FBS). The yellow and pink bars correspond to effective charge ratios *ρ*_eff_ = 4 and 10 on the lipoplex, respectively. The gray bar corresponds to Lipo2000*, used here as a positive control. The data represent the (mean ± SD) of three wells from three independent experiments.

In biological studies, gene nanovectors are not only required to be effective but also safe upon transfecting cells. Thus, once all the physicochemical factors characterizing the formation and stabilization of the LYCl/DOPE-pDNA lipoplexes were fully analyzed, it was interesting to see whether they would be capable of transfecting pDNAs into living cells and how efficient and cell-friendly they would be in doing so. For that purpose, a series of in vitro experiments on COS-7 cells were carried out. Figure 7a,b reports the cell transfection efficiency levels of LYCl/DOPE-pDNA lipoplexes at two molar compositions of the cationic lipid in the mixed lipids (α = 0.2 and 0.5) and different effective charge ratios of the lipoplex (*ρ*_eff_ = 4 and 10). Data were collected in terms of: (i) expressed %GFP (percentage of cells in which GFP expression was observed) and MFI (mean fluorescence intensity, i.e., average intensity of fluorescence per cell) for plasmid pEGFP-C3 (obtained from FACS experiments, panel (a); and (ii) ng of luciferase/mg of protein for plasmid pCMV-Luc VR1216 (obtained from luminometry measurements, panel (b). The experiments were performed in the presence of 10% serum (FBS) using Lipo2000* as the positive control. The corresponding cell viability responses are collected in Figure S17a,b of SM. 

The final aim of these experiments was to identify the values of α (percentage of LYCl and DOPE in the lipidic nanovector) and *ρ*_eff_ (charge ratio between the nanovector and plasmid in the lipoplex) that would afford the optimum formulations in vitro, which is crucial information for future in vivo applications. From a glance to Figure 7a,b, the transfection efficacy seems to depend on the plasmid to be transfected, the effective charge ratio of the lipoplex, and the molar fraction of the mixed lipid constituting the gene nanocarrier. As already reported for other lipoplexes containing pEGFP-C3 in the literature [49], the transfection efficiency is higher when the GCL/DOPE nanovector is composed mostly of DOPE (α = 0.2). This evidence is more pronounced in the case of pCMV-Luc (Figure 7b), where almost no transfection was observed at α = 0.5, confirming once again the important role of zwitterionic DOPE (neutral under experimental and/or physiological conditions) in transfection processes. In any case, it is interesting to notice that, the optimum lipid composition (α) in terms of transfection efficiency was found to be α = 0.5 in a previous work where cationic lipids (either gemini or monomeric), that incorporated an aromatic entity in their structures, were used to transfect p-EGFP-C3 plasmid to COS-7 cells [52]. This evidence comes to reinforce that the effect of DOPE content in the transfection results is very dependent on the structure and, mostly, charge density of the cationic lipid. It is true that its fusogenic character implies that the best transfection performances in many CL or GCL/DOPE systems have been found for mixtures with predominance in DOPE (low values of α), but it does not mean that all systems have to follow the same pattern. The cellular response is very complex and it is always convenient to test several compositions (α values) in order to choose the best one. Furthermore, in what respects to the lipoplex composition, it is remarkable that both *ρ*_eff_ = 4 and 10 afforded comparable transfection performances when transfecting the pEGFP-C3 plasmid while, in the case of pCMV-Luc, it is clear that *ρ*_eff_ = 10 is a better choice. 

In any case, all the amino acid-based formulations reported herein show better transfection levels in COS-7 cells than those of standard positive control Lipo2000* under equal experimental conditions, an interesting result in terms of efficacy improvement of non-viral vectors for gene delivery, given that Lipo2000* is one of the best controls used to compare the transfection efficacy. Furthermore, the cell viability results reported in Figure S17 show that all the LYCl/DOPE lipidic formulations transfect both plasmids (pCMV-Luc and pEGFP-C3) in a safe way, with viabilities exceeding 80% (normally considered the threshold level for cell safety). Notice that some formulations even exceed 90% viability. More interestingly, such low cytotoxicity found for LYCl/DOPE-pDNA formulations is comparable or even slightly better than that obtained for the control Lipo2000*. It seems that a lysine-derived moiety linked to a single hydrocarbon chain leads to much better performance of mixed lipid nanovectors as gene nanocarriers than two lysine moieties linked to a gemini lipid scaffold. In fact, it was not possible to transfect cells with gemini cationic lipid C_6_(LL)_2_/DOPE-pDNA lipoplexes [47] bearing two cationic lysine heads. This absence of transfection was attributed to the formation of ribbon structures that are known to prevent transfection. However, we have recently detected [46] certain levels of transfection in COS-7 cells when using a gemini cationic lipid with two histidine moieties on the heads, albeit lower than those reported herein. 

## 4. Conclusions

The incorporation of a lysine-derived residue to a C_12_ alkyl chain affords a biocompatible cationic lipid that, when mixed with the well-known fusogenic DOPE lipid, forms a non-viral gene nanocarrier able to stabilize, compact, and protect two different kinds of plasmids (encoding GFP and luciferase) by forming supramolecular LYCl/DOPE-pDNA lipoplexes. These lipoplexes are well organized in a L_α_ lamellar lyotropic liquid crystal phase characterized by a sandwich-type compaction pattern, with alternating bilayers of LYCl/DOPE mixed lipid (~4.5 nm width) and an aqueous monolayer containing the pDNA and counterions (~2 nm width). It is remarkable that both computational MD calculations and experimental results reveal that the generated lipid bilayers are stable at two different compositions of the LYCl/DOPE mixture, and that they are also capable of stabilizing and compacting fragments of DNA, which, if placed alone at that distance, would not interact or would interact repulsively (mainly electrostatic). Stabilization, as expected, is more favorable for the lipoplex with the highest LYCl content. The structural parameters obtained with the computational studies (d, d_m_ and d_w_) are in good agreement with those obtained from cryo-TEM and SAXS, and point (especially d_w_) to an efficient DNA compaction. In media containing living COS-7 cells, these gene nanocarriers were capable of crossing the cellular membrane and delivering the plasmid cargo in the cellular cytoplasm in a reasonably efficient and safe way. The best transfection outcomes were found when the mixed lipid was mostly constituted by DOPE (80% on a mole basis, i.e., α = 0.2). The transfection efficiency and cytotoxicity of the LYCl/DOPE nanocarriers are comparable or even better than those exhibited by the usual standard Lipo2000* employed in lipofection protocols. All these features point to these lipid-based gene nanovectors as a potentially interesting option for cell transfection in vitro, although transfection assays with other cell lines would be necessary to confirm it unequivocally. In any case, the results reported herein highlight the importance of designing and synthesizing gene delivery systems where different types of biocompatible residues are bound to a lipid colloidal matrix to increase the biochemical outcome in cellular environments and, as the final goal, in vivo.

## Figures and Tables

**Table 1:** Averaged values of distances shown in Figure 5 over the whole 1-ns MD trajectory (2000 structures).

Distance, nm	MD1	MD2	MD3
d_0_	8.8 ± 0.2	8.3 ± 0.1	8.4 ± 0.1
d_m_	4.6 ± 0.3	4.5 ± 0.2	4.6 ± 0.2
d_w_	2.0 ± 0.1	2.1 ± 0.1	2.1 ± 0.1
d	6.7 ± 0.2	6.4 ± 0.1	6.4 ± 0.1
d_1_	3.2 ± 0.1	2.8 ± 0.1	2.8 ± 0.1
d_2_	3.1 ± 0.2	3.1 ± 0.1	3.3 ± 0.1

## Supplementary Materials

The following are available online at https://www.mdpi.com/1999-4923/11/12/632/s1, Procedure for the determination of the effective charges of the cationic lipid LYCl and plasmid DNA; tables summarizing the data for the electrochemical and structural characterization; additional results of SAXS and cryo-TEM; theoretical protocols to build the tridimensional LYCl/DOPE bilayer (and the corresponding figures); the procedure for the lipid bilayer/DNA_2_ lipoplexes molecular dynamics simulation (and the corresponding figures); and cell viability outcomes.
