# Supplementary material for: A Non-Viral Plasmid DNA Delivery System Consisting on a Lysine-Derived Cationic Lipid Mixed with a Fusogenic Lipid
Source: Pharmaceutics. 2019 Nov 27;11(12):632. doi: 10.3390/pharmaceutics11120632 (PMC6956073; doi:10.3390/pharmaceutics11120632)
Supplement: Supplementary file 1 [file pharmaceutics-11-00632-s001.pdf]

pharmaceutics

MDPI

Supplementary Materials: A Non-Viral Plasmid DNA Delivery System Consisting on a Lysine-Derived Cationic Lipid Mixed with a Fusogenic Lipid

1. Determination of the effective charges of the cationic lipid (CL) and plasmid DNA (pDNA)

The composition of a mixed lipid used as gene vector is given as the molar fraction (α) of the cationic lipid, while in the case of the lipoplex, the composition may be given in terms of: a) the total lipid to DNA mass ratio, expressed as $m_L / m_{DNA} = (m_{L^+} + m_{L^0}) / m_{DNA}$, where m_L, m_{L^+}, m_{L^0}, and m_{DNA} are the mass of the total mixed lipid, CL, DOPE, and DNA, respectively, and b) the effective charge ratio (ρ_{eff}) expressed as the ratio between the charges of positive CL and negative DNA phosphate groups. All these quantities are related by the following two equations,

$$\alpha = \frac{m_{L^+} / M_{L^+}}{(m_{L^+} / M_{L^+}) + (m_{L^0} / M_{L^0})} \tag{1}$$

$$\rho_{eff} = \frac{n^+}{n^-} = \frac{q^+_{eff,\,L}(m_{L^+} / M_{L^+})}{q^-_{eff,\,DNA}(m_{DNA} / M_{DNA})} \tag{2}$$

where n^+ and n^- are the number of moles of positive and negative charges of CL and DNA; $q^+_{eff,\,L}$ and $q^-_{eff,\,DNA}$ are the effective charges of CL and pDNA per bp; and M_{L^+} and M_{DNA} are the molecular weight of the CL and pDNA per bp, respectively. There is a particular $(m_{L^+} + m_{L^0}) / m_{DNA}$ lipoplex composition where the positive and negative charges balance ($\rho_{eff} = 1$). This particular value, known as electroneutrality ratio $((m_{L^+} + m_{L^0}) / m_{DNA})_\phi$ is characteristic of the lipoplex and marks the lower limit from which the lipoplex becomes a potentially cell transfecting agent, since efficient cell transfection needs net positively charged lipoplexes capable of crossing the negatively charged cell membrane [1]. Zeta potential (ζ), related to the net charge of the lipoplex, is the best physicochemical property to determine this electroneutrality ratio. Figure S1 (panel a) of the MS shows plots of ζ vs. (m_L / m_{DNA}), at several α values for the CL used in this work. The electroneutrality ratio of the lipoplex can be determined as the m_L / m_{DNA} where a sign inversion on the charge on the ζ sigmoidal plots is detected. This value is related to the quantities of Eqs. (1-2) by,

$$\left(\frac{m_L}{m_{DNA}}\right)_\Phi = \left(\frac{m_{L^+} + m_{L^0}}{m_{DNA}}\right)_\phi = \frac{q^-_{DNA}[\alpha M_{L^+} + (1-\alpha) M_{L^0}]}{q^+_L \alpha M_{DNA}} \tag{3}$$

Studies reported in literature have shown that commercial linear DNA, as calf thymus DNA, or so on, has its negative charge totally available for the cationic lipid, i.e., $q_{linear\ DNA} = -2$ per base pair. But experiments reported by our group [2-5] have demonstrated that, at physiological conditions, plasmid DNA remains in a supercoiled conformation [6,7] rendering a much less negative charge than its nominal one $(q^-_{eff,\,pDNA} << -2/\text{bp})$. For that reason, the first step of any biophysical study must start with the determination of the effective charge of both, the cationic lipid $(q^+_{eff,\,L})$ and the pDNA $(q^-_{eff,\,DNA})$.

Initially, the effective charge of the CL $(q^{+}_{\text{eff, L}})$ can be determined for a certain CL composition (α) of the mixed lipid vector, from Eq. (3) using the $(m_L / m_{DNA})_{\Phi}$ value of the CL/DOPE-linear DNA lipoplex experimentally measured from zeta potential (black curve in Figure 2 of the MS), and assuming that $q_{\text{linear DNA}} = -2$/bp. Once the effective charge of the CL $(q^{+}_{\text{eff, L}})$ has been obtained, the determination of the electroneutrality value $(m_L / m_{DNA})_{\Phi}$ for the CL/DOPE-pDNA lipoplex containing the plasmid DNA in identical mixed lipid composition (α) (coloured curves in the Figure 2 of the MS) permits to obtain the effective charge of pDNA $(q^{-}_{\text{eff, DNA}})$ at each molar fraction of the mixed lipid (α) from rearranged Eq. (3) as follows,

$$q^{-}_{\text{eff,DNA}} = \left(\frac{m_L}{m_{DNA}}\right)_{\Phi} \left(\frac{q^{+}_{L^+} \alpha M_{DNA}}{\alpha M_{L^+} + (1-\alpha) M_{L^0}}\right) \tag{4}$$

Once the effective charges of the cationic lipid and plasmid DNA are correctly determined, the effective charge ratio (ρ_{eff}) of the lipoplex at any molar fraction (α) of the mixed lipid may be calculated by substituting $(q^{+}_{\text{eff, L}})$ and the pDNA $(q^{-}_{\text{eff, DNA}})$ in Eq. (2). The effective charge ratio of the lipoplex (ρ_{eff}) is the key to prepare lipoplexes with appropriate formulations to be used in biological studies, i.e., with a net positive charge.

Table S1. Values of electroneutrality ratio $(m_L / m_{DNA})_{\phi}$ for the LYCl/DOPE-DNA lipoplexes, at several molar compositions of the lipid mixture (α).

α	0.2	0.5	0.7
ctDNA	-	8.03	-
pDNA	3.00	1.02	0.48

Values estimated with a 5% error.

Table S2. Nominal and effective charges of the cationic lipid (CL) and pDNA.

α	0.2	0.5	0.7
$q^{+}_{\text{nom,L}}$	-	+1	-
$q^{+}_{\text{eff,L}}$	-	+0.45	-
$q^{+}_{\text{eff,L}} / q^{+}_{\text{nom,L}}$	-	+0.45	-
$q^{-}_{\text{nom,pDNA}} / \text{bp}$	-2	-2	-2
$q^{-}_{\text{eff,pDNA}} / \text{bp}$	-0.26	-0.25	-0.19
$q^{-}_{\text{eff,pDNA}} / q^{-}_{\text{nom,pDNA}}$	0.13	0.13	0.10
eff/ nom	3.5	3.6	4.7

Values estimated with an 8% error.

Table S3. Size (D_h) and polydispersities (PDI) of LYCl/DOPE-pDNA lipoplexes at two effective charge ratios (ρ_{eff}) and at two molar compositions of the mixed lipids (α).

α = 0.2	ρ_{eff} = 4		ρ_{eff} = 10	
	D_h (nm)	PDI	D_h (nm)	PDI
LYCl/DOPE- pEGFP-C3	173	0.07	152	0.14
LYCl/DOPE-pCMV-Luc	147	0.19	163	0.15
α = 0.5				
LYCl/DOPE- pEGFP-C3	210	0.30	174	0.24
LYCl/DOPE-pCMV-Luc	229	0.18	163	0.15

Errors are less than 10%.

Table S4. Values of the periodic distance of the lamellar structure (d) and pDNA-pDNA correlation distance for LYCl/DOPE-pDNA lipoplexes at several effective charge ratios (ρ_{eff}) and molar compositions (α). Values of q_{hkl} and d are in nm^{-1} and nm, respectively.

ρ_{eff}		α			
		**0.2**	**0.4**	**0.5**	**0.7**
1.5	q_{100}	1.0	1.0	1.0	1.1
	d_{100}	6.5	6.5	6.0	5.7
	q_{pDNA}	1.1	1.3	1.4	1.5
	d_{pDNA}	5.7	4.8	4.4	4.3
2.5	q_{100}	0.9	1.0	0.9	1.1
	d_{100}	7.2	6.5	7.0	5.8
	q_{pDNA}	1.2	1.5	1.3	1.4
	d_{pDNA}	5.2	4.2	4.7	4.4
4.0	q_{100}	0.9	0.9	0.9	1.0
	d_{100}	7.1	7.1	7.0	6.3
	q_{pDNA}	1.5	1.4	1.4	1.5
	d_{pDNA}	4.3	4.5	4.4	4.1

Values of q_{hkl} and d are estimated with a 5% and 10% error respectively.

(a)

(b)

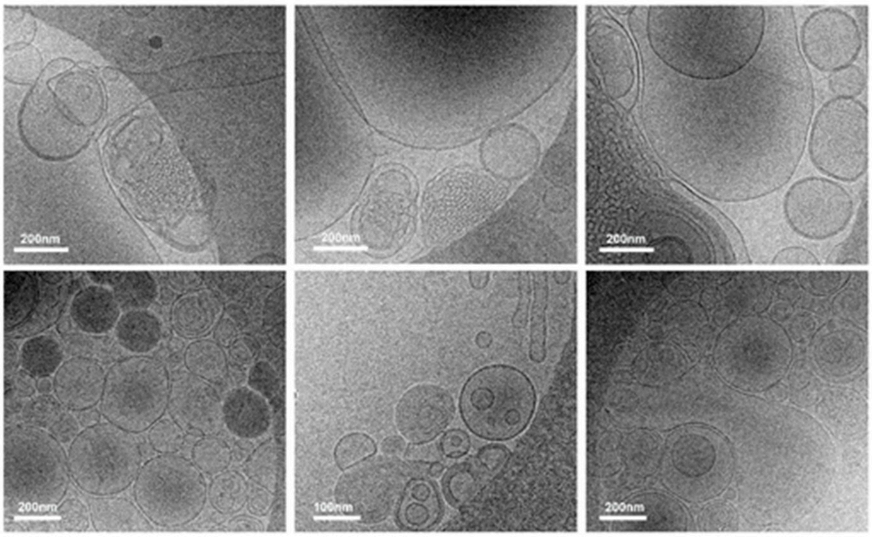

Figure S1. A selection of cryo-TEM micrographs of LYCl/DOPE-pDNA lipoplexes at ρ_{eff} = 4 and at molar compositions of the cationic lipid in the mixed lipids of α = 0.2 (**a**) and α = 0.5 (**b**).

2. Theoretical protocols for building a tridimensional (3D) LYCl/DOPE bilayer

1) Interaction of two neutral DOPE units by the hydrophobic tails. Initially two neutral optimized extended conformations of DOPE units were approached along the y coordinate. For this purpose a DOPE unit was fixed in a coordinate system, as depicted in Figure S2, and a second DOPE was approached step by step in 0.5 Å intervals along the y coordinate from y = +50 to +15 (Å) in the presence of water. Previously, the most favorable relative DOPE-to-DOPE orientation (torsional angle, θ) for the approaching was estimated from the DOPE-DOPE interaction energies obtained by changing the lipid distance along the y coordinate and the θ angle in small steps. Figure S2 depicts the most probable orientation for the approaching. Figure S3 shows results of the total binding energy and electrostatics and van der Waals contributions as a function of the *oo'* distance obtained during the approaching of DOPE units with the most favorable orientation, along the y coordinate. Total binding energies were negative and they decreased with the distance up to reaching a MBE (minimum binding energy) structure. The larger contributions came from van der Waals interactions. Figure S3 also depicts the MBE one-dimensional dimer structure (named DOPEDIM). This structure, once the water molecules were removed, was employed in the next step.

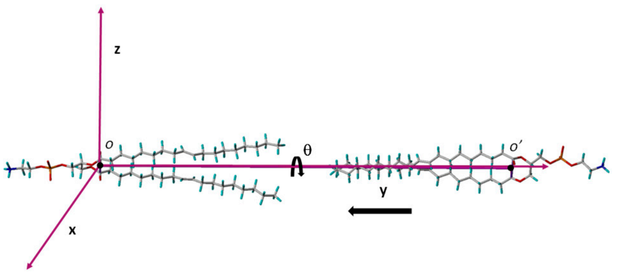

Figure S2. Coordinate system used for DOPE-DOPE approaching along the y coordinate.

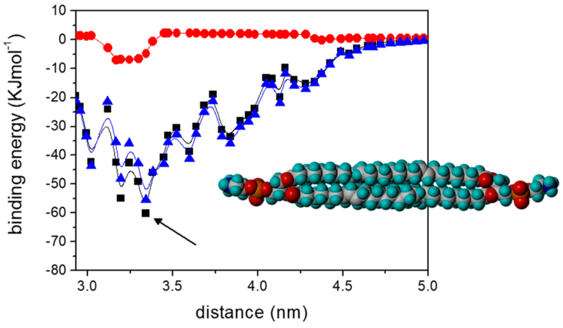

Figure S3. Total binding energy (black squares) and electrostatics (red circles) and van der Waals (blue triangles) contributions upon DOPE-DOPE approaching along the y coordinate with the optimized orientation. The distance measured is between *o* and *o′*. Superimposed is the MBE dimer structure (named DOPEDIM) indicated by the arrow.

2) Lateral DOPEDIM dimers interaction. Two MBE DOPE dimer structures (1 and 3) were approached to a third one (2), as shown in Figure S4, along the z coordinate from z = +15 to +5 (Å) (or z = -15 to -5 Å) in 0.25 Å intervals in the presence of water. Figure S5 shows the total binding energy and non-bonded contributions as a function of the d_{12} and d_{23} average distances obtained during the approaching of the 1 and 2 DOPEDIM dimers with the most favorable orientations, as depicted in Figure S4. Binding energies were negative and they decreased with the distance up to a MBE (minimum binding energy) structure is reached. Figure S5 also depicts the MBE structure indicated by the arrow. For this structure (named 3DOPEDIM) the d_{12} and d_{23} distances were 0.8 and 0.82 nm respectively (average distance 0.81 nm).

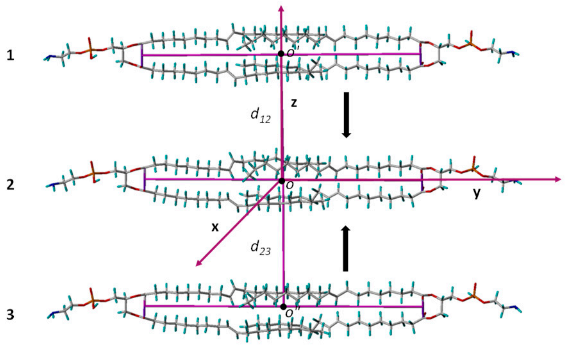

Figure S4. Coordinate system used for three DOPE dimers (DOPEDIM) which approach each other laterally along the z coordinate.

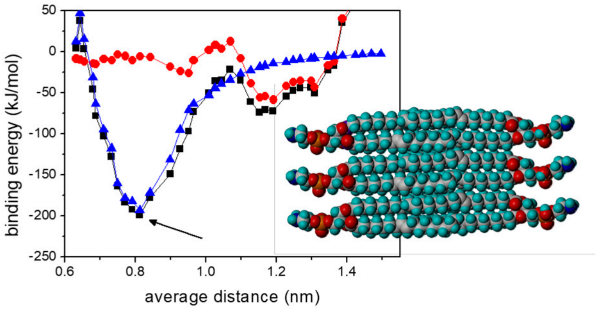

Figure S5. Total binding energy (black squares) and electrostatics (red circles) and van der Waals (blue triangles) contributions upon two (1 and 3) DOPE dimer structures (DOPEDIM) approaching dimer 2 along the z coordinate with the optimized orientation. The average distance was of the d_{12} and d_{23} distances. Superimposed is the MBE structure (named 3DOPEDIM) indicated by the arrow.

3) Interaction of 4 LYCl lipid units with a DOPEDIM dimer. In a similar manner, 4 charged (+1 esu, each) LYCl units were simultaneously approached to the DOPEDIM dimer as depicted in Figure S6. Such approaching was performed from z = +15 to +3 (Å) (or z = -15 to -3 (Å) in 0.25 Å steps. Figure S7 shows the results of the total binding or interaction energy and non-bonded contributions as a function of the average of the four d_1, d_2 d_3 and d_4 distances along the z coordinate represented in Figure S6. As expected, total binding energies were favorable upon approaching. Van der Waals is the most important contribution, becoming more negative as the distances decrease, again reaching a MBE structure (see arrow), which is depicted in the same Figure S7. For this structure the d_1, d_2 d_3 and d_4 distances were 5.4, 5.2, 5.3 and 5.3 Å respectively (average 5.3 Å). This structure, named 4LYClDOPEDIM was used in the next step.

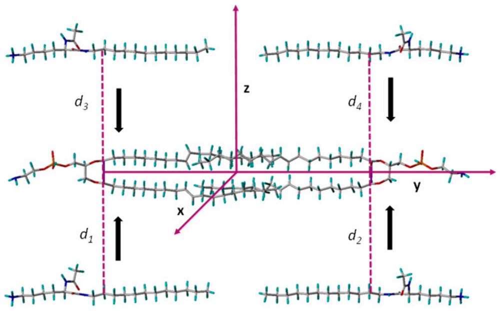

Figure S6. Coordinate system used for the simultaneous approaching of four LYCl units to the DOPE dimer along the z coordinate.

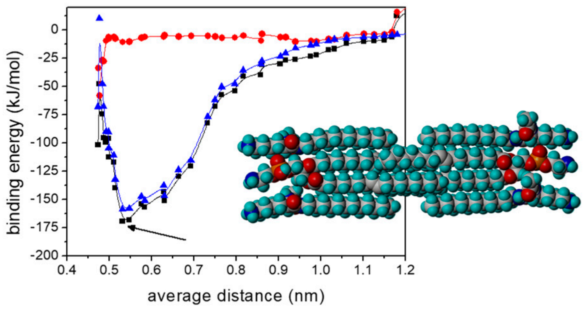

Figure S7. Total binding energy (black squares) and electrostatics (red circles) and van der Waals (blue triangles) contributions upon two (1 to 4) LYCl units simultaneously approaching DOPEDIM

dimer 2 along the z coordinate with the optimized orientation. The distance measured (along z axis) is the average of the four distances depicted in Figure S6. Superimposed is the MBE structure indicated by the arrow.

4) Interaction of two DOPEDIM dimers with the 4LYClDOPEDIM complex (generated previously in step 3). Two DOPEDIM dimers were simultaneously approached along the z coordinate from the positive and negative sides to the 4LYClDOPEDIM MBE structure depicted in Figure S7. Such approaching was performed from z = +10 to +4 (Å) (or z = -10 to -4 (Å) in 0.25 Å steps, as Figure S8 shows.

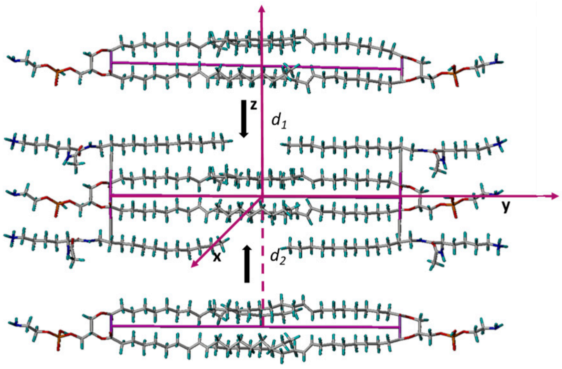

Figure S8. Coordinate system used for the simultaneous approaching of two DOPEDIM dimers to the 4LYClDOPEDIM structure generated in step 3 along the z coordinate.

Figure S9 shows the results of the total binding or interaction energy and non-bonded contributions as a function of the average of d_1 and d_2 distances along the z coordinate represented in Figure S8. The total binding energy is favorable except for the shortest distances. Van der Waals is the most important contribution, becoming more negative as the distance decrease. It then reaches a MBE structure, which is depicted in Figure S9. Electrostatics are also favorable during approaching. For this structure, the d_1 and d_2 distances were 5.7 and 5.9 Å respectively (average 5.8 Å). This structure, named 4LYCl3DOPEDIM, was used, in the next steps, for building a 3-dimensional LYCl/DOPE bilayer.

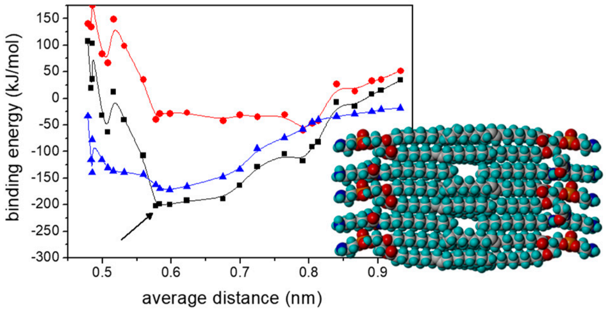

Figure S9. Total binding energy (black squares) and electrostatics (red circles) and van der Waals (blue triangles) contributions upon the simultaneous approaching of two DOPEDIM dimers to the MBE structure, 4LYClDOPEDIM, along the z coordinate. The distance measured is the average of both d_1 and d_2 distances depicted in Figure S8. Superimposed is the MBE structure indicated by the arrow (named, 4LYCl3DOPEDIM).

5) Lateral interaction of two 3DOPEDIM structures with the two-dimensional 4LYCl3DOPEDIM structure (generated previously in step 4). Two 2D 3DOPEDIM structures close to the MBE ones, depicted in Figure S5 (3DOPEDIM), were simultaneously approached along the x coordinate to the

MBE two-dimensional 4LYCl3DOPEDIM structure (Figure S9) from the positive and negative sides, as depicted in Figure S10. Such approaching was performed from x = +20 to +5 (Å) (and x = -20 to -5 (Å) in 0.25 Å intervals. As Figure S11 shows, the total interaction energies were favorable upon approaching. Van der Waals is the most important contribution becoming more negative as decreasing the distances. Electrostatics exhibit an energy barrier at distances smaller than ~1 nm which is mainly due to DOPE units lateral interaction between DOPEDIM 2D structures. At smaller distances than ~1 nm, the total energy also significantly increases (as well as the strain energy). The structure indicated by the arrow is the one for which van der Waals interactions are favorable and the electrostatics interactions begin to become repulsive. This structure is also the limit one for which the total energy begins to become quite large. This 3D structure, named 4LYCl/9DOPEDIM once optimized, was one of the ones used to interact with DNA fragments as described in the main text of this manuscript. It is remarkable, that this structure which consists of approximately 18% LYCl and 82% DOPE, from a molecular point of view, may be considered as a fragment of a mixed lipid bilayer of similar composition to that used in the experiments.

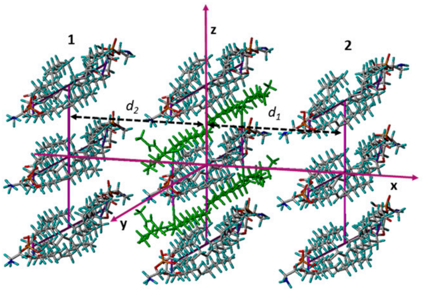

Figure S10. Coordinate system used for the simultaneous approaching of two 3DOPEDIM structures along the x coordinate to the two-dimensional structure generated in the previous step 4, 4LYCl3DOPEDIM.

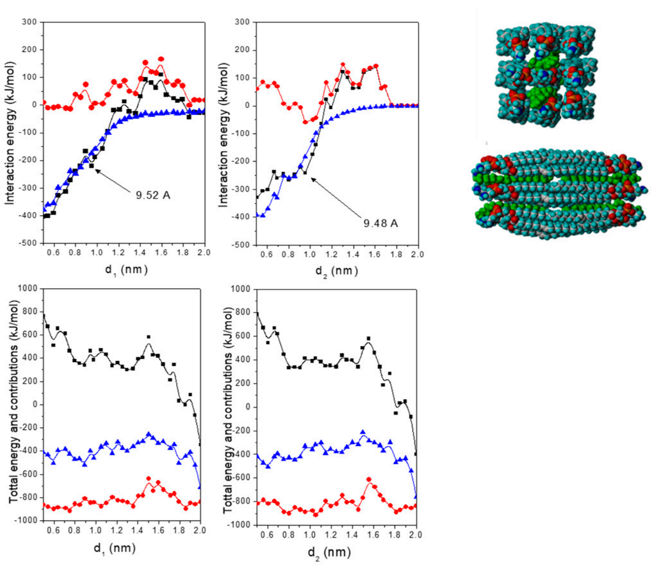

Figure S11. (Upper panels) Total binding energy (black squares) and electrostatics (red circles) and van der Waals (blue triangles) contributions upon the simultaneously approaching two 3DOPEDIM 2D structures to the 4LYCl3DOPEDIM MBE structure along the x coordinate. Interaction energies are between each 3DOPEDIM structure 1 (left) and 2 (right) and the central 4LYCl3DOPEDIM. The distances measured are d_1 and d_2, depicted in Figure S10. (Bottom panels) Idem for total energy (black squares) and electrostatics (red circles) and van der Waals (blue triangles) contributions. Two view of one of the most probable structures of the tree-dimensional lipidic bilayer indicated by the arrow (named, 4LYCl/9DOPEDIM) are depicted.

6) Lateral interaction of two 3DOPEDIM to which 4 LYCl units were added, with the 4LYCl3DOPEDIM structure (generated in step 4). Two 2D structures, 1 and 2 (Figure S12), which were built from each of a 3DOPEDIMs and 4 LYCl (placed at the optima distances obtained for the MBE structure), were simultaneously approached along the x coordinate from the positive and negative sides to the 2D 4LYCl3DOPEDIM MBE structure (depicted in Figure S9). This approaching was also performed from x = +20 to +5 (Å) (or x = -20 to -5 (Å) in 0.25 Å steps as Figure S12 shows. As represented in Figure S13, the total interaction energies were favorable upon approaching. Van der Waals is the most important contribution, becoming more negative as the distances decrease. Electrostatics, however, exhibit an energy barrier due to the unfavorable interaction between the 1 and 2 structures and the 4LYCl3DOPEDIM one. Something similar, not showed, occurs with the total energy. The structure indicated by the arrow, at distances where van der Waals interactions are favorable and a slight minimum was obtained for the electrostatics interactions, is depicted in Figure S13. This 3D structure, named 12LYCl/9DOPEDIM, once optimized, was the other one used to interact with DNA fragments, as was also described in the main text of this manuscript. Notice that this other fragment of the 3D lipidic bilayer consists of near 40% cationic LYCl and 60% zwitterionic DOPE, which means a significant different molecular content that the previous generated 4LYCl/9DOPEDIM bilayer.

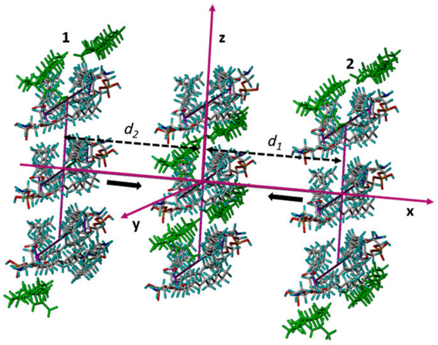

Figure S12. Coordinate system used for the simultaneous approaching of two 3DOPEDIM 2D structures to which 4 LYCl were added (named 1 and 2), along the x coordinate to the 4LYCl3DOPEDIM 2D structure generated in step 4.

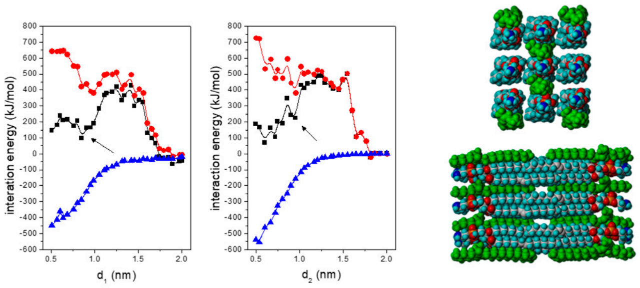

Figure S13. Total binding energy (black squares) and electrostatics (red circles) and van der Waals (blue triangles) contributions upon the simultaneously approaching two 3DOPEDIM+4LYCl 2D structures to the 4LYCl3DOPEDIM MBE structure along the x coordinate. Interaction energies are between each, 1 (left) and 2 (right), structure and the central 4LYCl3DOPEDIM unit. The distances measured are d_1 and d_2 depicted in Figure S12. Superimposed is one of the most probable structures of the 3D system indicated by the arrow (named, 12LYCl/9DOPEDIM).

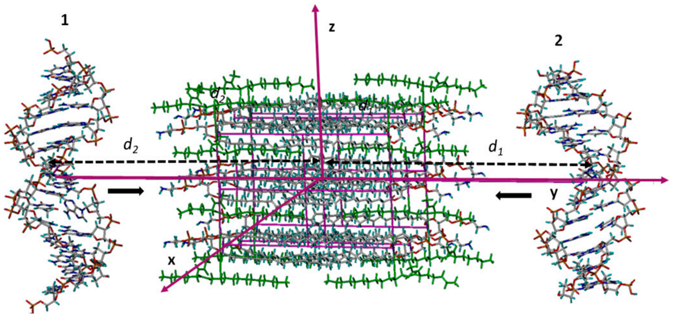

Figure S14. Coordinate system used for the two DNA fragments simultaneously approaching the 12LYCl/9DOPEDIM cationic bilayer by the major groove along the *y* coordinate. The coordinate axis and some "dummy" bonds are drawn in magenta.

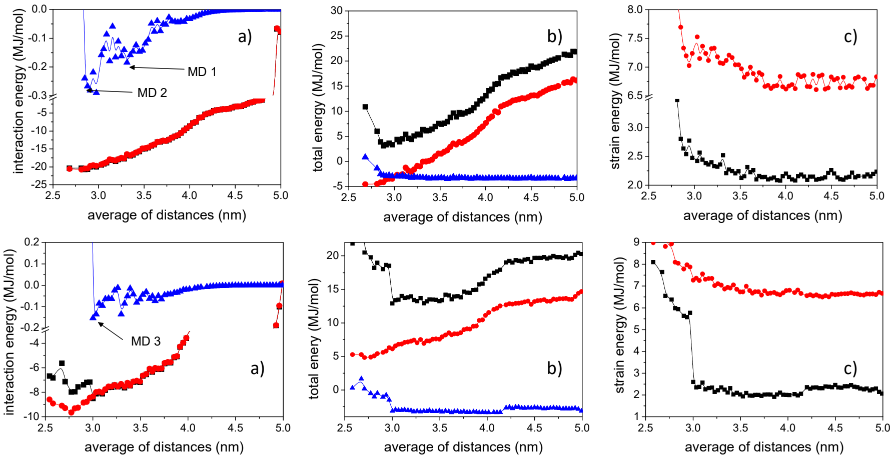

Figure S15. (a) Total interaction energy (black) and electrostatics (red) and van der Waals (blue) contributions between DNA fragments and the 3D lipid bilayers; (b) total energy and contributions (same colors) and (c) strain energy (sum of bond stretching, angle bending and torsional energies) for 3D lipid bilayers (black) and DNA fragments (red). The coordinate x corresponds to the average of d_1 and d_2 distances depicted in Figure S14. The upper panels are the results for (12LYCl/9DOPEDIM)(DNA)$_2$ lipoplex and the bottom panels for the (4LYCl/9DOPEDIM)(DNA)$_2$ ones.

3. Procedures for the lipid bilayers/DNA$_2$ lipoplexes Molecular Dynamics simulation.

In brief: 1ns MD trajectories were performed in in the presence of explicit water. Bonds where H atoms were involved were constrained from vibrating, but the rest of the conformational parameters were variable. MD trajectories were performed starting from 1 K, and the temperature was increased by 20 K intervals equilibrating the system at each intermediate temperature for 500 fs up to reaching the temperature of interest of 300K. Once at this temperature, an additional equilibration period up to 25 ps was used. The whole heating/equilibration period was discharged from the analysis. From this point, the rest of the 1ns trajectory was simulated at 2fs integration time steps. The velocities were rescaled at 10 fs intervals. Structures obtained from the analysis of MD trajectories were saved every 500 fs, yielding 2000 images for subsequent analysis. The averages of any property obtained from the MD trajectory analysis was calculated by equally weighing each image. To maintain a regular helical structure for the pair of DNA chains avoiding the unwinding of the end portions of the short DNA helix fragments during MD, N…HN hydrogen bond distances for each pair of DNA pair of complementary bases were constrained to keep them constant. Then, a harmonic penalty function was added to the force field equation for those atoms which were involved

in the constraint. This energy function was written as $E = k\,(r - r_i)^2$, where $k = 200$ kcal/molÅ^2, r_i and r are the initial distance and the variable distance during the MD trajectory, respectively.

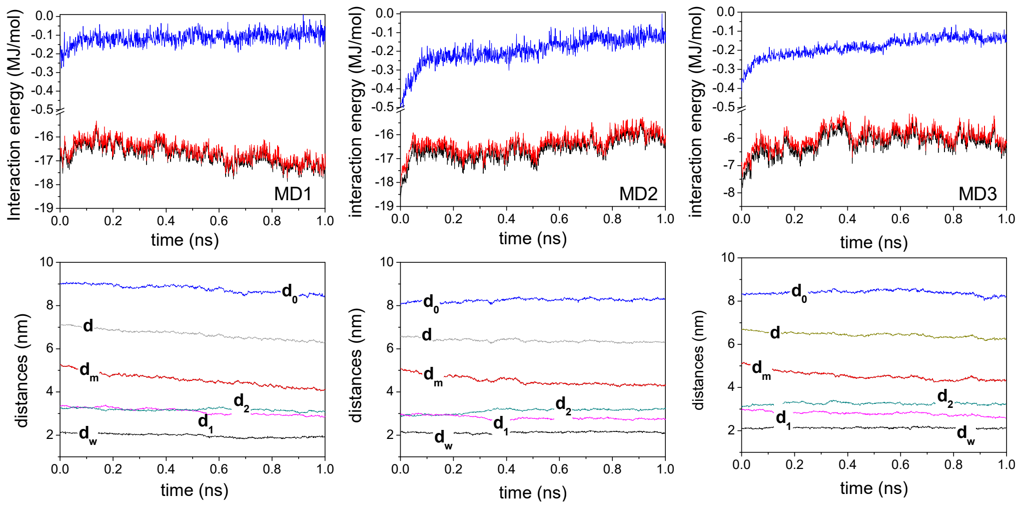

Figure S16. (Upper panels) Histories of the total interaction energy (black) and electrostatics (red) and van der Waals contributions (blue) between $(DNA)_2$ fragments and lipid bilayers, obtained from the analysis of the 1ns MD trajectories in the presence of water on the optimized MD1 and MD2 structures for $(12LYCl/9DOPEDIM)(DNA)_2$ lipoplex and MD3 for $(4LYCl/9DOPEDIM)(DNA)_2$ one. The average of these structures along the whole trajectory is depicted in Figure 4. (Bottom panels) Histories for several distances (see Figure 5) obtained from the analysis of MD simulations. These distances are defined as: d_0 is measured as the distance (along the y coordinate) between the center of mass of oxygen atoms bounded to P atoms of phosphate units 4 and 8′ of DNA1 fragment and the center of mass of oxygen atoms for the same phosphate units of the opposite DNA2 fragment; d_m is measured as the distance (along the y coordinate) of center of mass of N atoms located at one side and the center of mass of N atoms located at the other side of DOPEDIMER units of the bilayer; d_w is calculated as the distance (along the y coordinate) of center of mass of oxygens bounded to P atoms of 4 and 8′ phosphate units and the center of mass of oxygen atoms bounded to P atoms of 8 and 4′ phosphate units of each DNA fragment (for each structure the value is the average for both DNA units); d (periodic distance) is measured as the distance (along the y coordinate) between the center of mass of N terminal atoms located at one side of the DOPEDIMER units of the bilayer and center of mass of oxygens bounded to P atoms of phosphate units 4 and 8′ for the farthest DNA unit (for each structure the value is the average for both d distances); d_1 and d_2 are the distances (along the y coordinate) between the center of mass of each DNA fragments and the origin of the coordinate system.

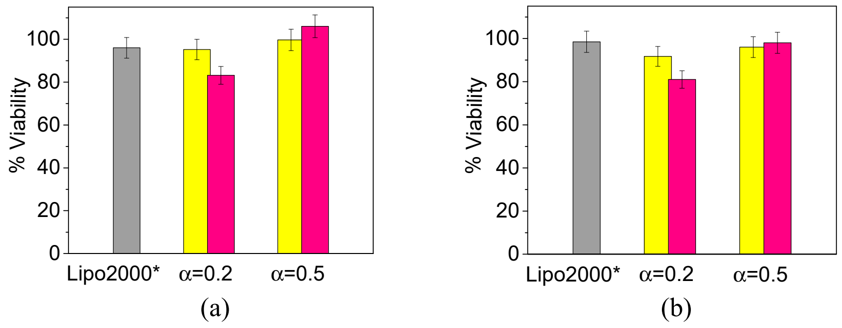

Figure S17. Cell viability of COS-7 cells in the presence of LYCl/DOPE-pDNA lipoplexes at two molar compositions of the cationic lipid in the mixed lipids (α = 0.2 and 0.5). The plasmids used were (**a**) pEGFP-C3 and (**b**) pCMV-Luc VR1216. The results were normalized to those obtained for untreated cells (100%). All the experiments were performed with 10% serum (FBS). The yellow and pink bars correspond to effective charge ratios ρ_{eff} = 4 and 10 on the lipoplex, respectively. The gray bar corresponds to Lipo2000*, used here as a positive control. The data represent the (mean ± SD) of three wells from three independent experiments.

References

1. Dias, R.S.; Lindman, B. *DNA Interaction with Polymers and Surfactants*; Wiley & Sons: Hoboken, NJ, 2008; 10.1371/journal.pone.0092692.
2. Muñoz-Úbeda, M.; Misra, S.K.; Barrán-Berdón, A.L.; Aicart-Ramos, C.; Sierra, M.B.; Biswas, J.; Kondaiah, P.; Junquera, E.; Bhattacharya, S.; Aicart, E. Why is less cationic lipid required to prepare lipoplexes from plasmid DNA than linear DNA in gene therapy? *J. Am. Chem. Soc.* **2011**, *133*, 18014-18017, doi:10.1021/ja204693f.
3. Barrán-Berdón, A.L.; Misra, S.K.; Datta, S.; Muñoz-Úbeda, M.; Kondaiah, P.; Junquera, E.; Bhattacharya, S.; Aicart, E. Cationic gemini lipids containing polyoxyethylene spacers as improved transfecting agents of plasmid DNA in cancer cells. *J. Mater. Chem. B* **2014**, *2*, 4640-4652, doi:10.1039/C4TB00389F.
4. Martínez-Negro, M.; Guerrero-Martínez, A.; García-Río, L.; Domènech, O.; Aicart, E.; Tros de Ilarduya, C.; Junquera, E. Multidisciplinary approach to the transfection of plasmid DNA by a nonviral nanocarrier based on a gemini–bolaamphiphilic hybrid lipid. *ACS Omega* **2018**, *3*, 208-217, doi:10.1021/acsomega.7b01657.
5. Misra, S.K.; Muñoz-Úbeda, M.; Datta, S.; Barrán-Berdón, A.L.; Aicart-Ramos, C.; Castro-Hartmann, P.; Kondaiah, P.; Junquera, E.; Bhattacharya, S.; Aicart, E. Effects of a delocalizable cation on the headgroup of gemini lipids on the lipoplex-type nano-aggregates directly formed from plasmid DNA. *Biomacromolecules* **2013**, *14*, 3951-3963, doi:10.1021/bm401079h.
6. Lyubchenko, Y.L.; Shlyakhtenko, L.S. Visualization of supercoiled DNA with atomic force microscopy in situ. *Proc. Natl. Acad. Sci. U.S.A.* **1997**, *94*, 496-501, doi:10.1073/pnas.94.2.496.
7. Foldvari, M.; Badea, I.; Wettig, S.; Verrall, R.; Bagonluri, M. Structural characterization of novel gemini non-viral DNA delivery systems for cutaneous gene therapy. *J. Exp. Nanosci.* **2006**, *1*, 165-176, doi:10.1080/17458080500411965.
